# Whole genome sequencing and molecular epidemiology of methicillin-resistant *Staphylococcus aureus* isolated from patients with bacteraemia in Slovenia

**DOI:** 10.1007/s10096-024-04802-1

**Published:** 2024-03-22

**Authors:** Urška Dermota, Andraž Celar Šturm, Tina Triglav, Katja Strašek Smrdel, Ivana Velimirović

**Affiliations:** Institute of microbiology and immunology, Faculty of Medicine, University of Ljubljana, Zaloska 4, Ljubljana, 1000 Slovenia

**Keywords:** MRSA, Bloodstream infections, Whole genome sequencing, Slovenia

## Abstract

**Purpose:**

Data on the molecular epidemiology of methicillin-resistant *Staphylococcus aureus* isolates from patients with bacteraemia in Slovenia are lacking. The aim of this study was to phenotypically and genotypically investigate 82 MRSA strains isolated from patients with bloodstream infections in central Slovenia between 2019 and 2022.

**Methods:**

Whole-genome sequencing of selected strains was performed to characterize the strains based on sequence typing, antimicrobial resistance, toxin, and virulence factors genes.

**Results:**

Most MRSA carried SCC*mec* II (63.4%), followed by SCC*mec* IV (34.1%) and SCC*mec* V (2.5%). A high proportion of strains belonging to the ST225 lineage (45.1%) was observed, followed by ST97 (18.3%), ST2883 (15.9%), ST22 (9.8%), ST5 (3.7%), and the ST1, ST398 and ST45 lineages (2.4% each). Sixteen different *spa* types were identified, predominantly ST225-t003 (31.7%), ST97-t359 (15.9%), and ST2883-t4336 (14.6%). None of the strains carried Panton-Valentine leukocidin, exfoliative toxins, or toxic shock toxin. All MRSA strains were susceptible to linezolid, rifampicin, vancomycin, teicoplanin, and trimethoprim-sulfamethoxazole. MRSA strains were resistant to erythromycin, clindamycin, tetracycline and gentamicin, with a frequency of 74.4%, 74.4%, 8.5%, and 1.2%, respectively.

**Conclusion:**

This study demonstrates that bacteraemia in central Slovenia is caused by diverse MRSA lineages. Identification of newly emerged lineages should be followed in the future to detect changes in the molecular epidemiology of MRSA in our country.

**Supplementary Information:**

The online version contains supplementary material available at 10.1007/s10096-024-04802-1.

## Introduction

The global epidemiology of methicillin-resistant *Staphylococcus aureus* (MRSA) has changed due to different sources of MRSA strains, including hospital-associated MRSA (HA-MRSA), community-associated MRSA (CA-MRSA), and livestock-associated MRSA (LA-MRSA) worldwide. Epidemiologic distinctions between HA-MRSA, CA-MRSA, and LA-MRSA are blurred because MRSA clones circulate in hospital settings, communities, and livestock environment [1, 2].

MRSA is an important nosocomial pathogen that causes bloodstream infections (BSIs), which significantly increase morbidity and mortality, antibiotic use, and the cost of treatment [1, 3, 4]. In the United States, the USA300 clone is the most common strain causing nosocomial MRSA bacteraemia, whereas in Europe, most nosocomial MRSA infections continue to be caused by HA-MRSA genotypes (e.g. ST5, ST239, ST247, ST250, ST22, ST45), except in countries with industrial swine production, where LA-MRSA ST398 is predominant [1, 2, 5–8].

MRSA is well controlled in Slovenian hospitals, but unfortunately the molecular epidemiology of MRSA in Slovenia is poorly documented, even if the molecular epidemiology of MRSA in Europe has been extensively documented [6–8]. At the national level, we only have data on antimicrobial resistance and prevalence of MRSA isolates from the Slovenian National Antimicrobial Susceptibility Testing Committee (SKUOPZ) and from the European Resistance Surveillance Network (EARS-Net). According to the data from EARS-Net, the prevalence of invasive MRSA isolates from blood cultures or cerebrospinal fluid among all *S. aureus* isolates in Slovenia in 2021 was 7.8% [9]. According to latest available data from SKUOPZ the prevalence of MRSA isolates from clinical samples among all *S. aureus* isolates in Slovenia in 2017 was 7.7% [10].

In addition, limited data on virulence gene profiles and clonality of *S. aureus* and MRSA have been reported in Slovenia [11–15]. There are very few published reports investigating the molecular epidemiology of MRSA bloodstream infections in Slovenia. To our knowledge, the last publication on the genetic characterization of 10 invasive MRSA isolates from blood cultures is from 2006, and the predominant *spa* type was t041 [16, 17].

For these reasons, we performed whole-genome sequencing of MRSA isolates recovered from blood cultures between 2019 and 2022 in central Slovenia to investigate the dominant MRSA genotypes causing bloodstream infections and to determine their antimicrobial resistance genes and virulence factors.

## Materials and methods

### Settings

Slovenia has an area of 20,273 km^2^ and a population of 2 approximately million. The Department of Bacteriology of the Institute of Microbiology and Immunology of the Faculty of Medicine, University of Ljubljana (IMI) serves three hospitals and covers a central region of about 700 000 inhabitants.

### Collection of bacterial strains

All non-duplicated MRSA strains isolated from blood cultures of individual patients between January 2019 and December 2022 at IMI, were included in our retrospective study. IMI serves three hospitals and processes approximately 38 000 blood cultures per year, using the BD Bactec FX400 (BD, Sparks, USA) according to the manufacturer’s instructions. Blood cultures were incubated for 5 days, and all positive bottles were processed according to the laboratory’s standard operating procedures, which include Gram staining, subculture, identification, and antimicrobial susceptibility testing.

### Identification of bacteria and phenotypic antimicrobial susceptibility testing

*S. aureus* isolates were identified by MALDI-TOF mass spectrometry (Bruker Daltonic GmBH, Bremen, Germany), and MRSA isolates were detected by antibiotic susceptibility testing using the agar disk diffusion method according to the European Committee on Antimicrobial Susceptibility Testing (EUCAST) [18]. The antibiotics tested were penicillin, cefoxitin, gentamicin, erythromycin, clindamycin, tetracycline, ciprofloxacin, trimethoprim-sulfamethoxazole, rifampin, linezolid, and mupirocin (BD, Sparks, USA). The minimal inhibitory concentration (MIC) of vancomycin and teicoplanin was determined using the E-test (bioMerieux, Marcy-l’Etoile, France).

### Patient data

For all patients with MRSA-positive blood cultures, data on gender, age, and date of sample collection were extracted from the MBL laboratory information system (SRC Infonet, Kranj).

### Whole genome sequencing

Whole genome sequencing was performed on 82 isolates. DNA was extracted using the DNeasy blood and tissue kit (Qiagen), and genomic libraries were prepared with Nextera XT Library Preparation Kit (Illumina). Isolates were sequenced, targeting a minimum coverage of 150x with the NextSeq 550 System (Illumina) using 2 × 149 bp paired-end reads chemistry. Fastp v0.23.2 [19] was used to trim the raw reads of adapter sequences and low quality reads. Quality of both raw and trimmed reads was assessed with FastQC v0.11.9 [20]. Assembly of trimmed reads into contigs was done with SPAdes v3.15.3 [21] using the default kmer values and “--careful” parameters. Quast v5.2.0 [22] was used for quality assessment of the assemblies.

### MLST, cgMLST, *spa* and SCC*mec* typing

Sequence types of isolates were determined in silico using the *S. aureus* typing database on PubMLST [23]. SpaTyper v0.3.3 [24] was used for typing of isolates, using the repeat sequences and repeat orders available on the spa typing website (https://spa.ridom.de/index.shtml) [25]. Staphylococcal chromosomal cassette typing was performed using SCCmecFinder v1.2 [26] with default parameters. Phylogenetic analysis of isolates was performed with core genome MLST, using the typing scheme for *S. aureus* available in Ridom SeqSphere+ [27]. A neighbor joining tree (NJT) was constructed based on the cgMLST allele differences, ignoring pairwise missing values and using only samples with more than 95% of the 1861 available alleles. The iTOL online phylogenetic tree display tool was used to visualize and annotate the tree [28].

### Virulence factors and antimicrobial resistance genes

Antimicrobial resistance genes present in isolates were identified with ResFinder 4.1, using default parameters [29]. Virulence factors were identified with ABricate v1.0.1 [30] with default settings, using the virulence factor database (VFDB) [31], as well as the AlereMicroarray_Virulence tool available in Ridom SeqSphere + software [32].

### Data availability

The generated raw reads of all isolates were submitted to the European nucleotide archive under the study accession number PRJEB66124.

## Results

Between 2019 and 2022, a total of 1023 nonrepeating *S. aureus* isolates were identified in patients with bloodstream infections. Eighty-two (8.0%) were characterized as MRSA and included in our study.

### Molecular epidemiology

All identified MRSA carried the *mec*A gene; none of the isolates carried the *mec*C gene. The MRSA blood culture isolates belonged to six different clonal complexes (CC) and to eight different sequence types (ST). The most prevalent CC was CC5 (64.7%, *n* = 53), followed by CC97 (18.3%, *n* = 15), CC22 (9.8%, *n* = 8), CC1 (2.4%, *n* = 2), CC45 (2.4%, *n* = 2) and CC398 (2.4%, *n* = 2). The most prevalent ST was ST225 (45.1%, *n* = 37), followed by ST97 (18.3%, *n* = 15), ST2883 (15.9%, *n* = 13), ST22 (9.8%, *n* = 8), ST5 (3.7%, *n* = 3), ST1 (2.4%, *n* = 2), ST45 (2.4%, *n* = 2) and ST 398 (2.4%, *n* = 2). Genetic diversity of MRSA isolates was determined using *spa* typing. A total of 16 *spa* types were observed, and the most predominant type was t003 (31.7%, *n* = 26). Two isolates were non-typable by *spa* typing. In SCC*mec* typing, three types (II, IV and V) were determined among the 82 MRSA isolates. The most frequently confirmed type was SCC*mec* II (63.4%, *n* = 52), followed by SCC*mec* IV (34.1%, *n* = 28) and SCC*mec* V (2.5%, *n* = 2). With *agr* typing, three types (I, II and III) were determined. 32.9% (*n* = 27) MRSA carried *agr* I, 64.6% (*n* = 53) carried *agr* II in and 2.5% (*n* = 2) *agr* III.

### Phenotypic and genotypic antimicrobial resistance

All 82 MRSA isolates carried the *mec*A gene and were phenotypically resistant to cefoxitin. Antimicrobial susceptibility results showed that all isolates were fully susceptible to several antibiotics tested: linezolid, rifampicin, vancomycin, teicoplanin and trimethoprim-sulfamethoxazole.

91.5% of isolates (*n* = 75) carried the *bla*Z gene encoding a penicillinase, while 8.5% (*n* = 7) of MRSA isolates, belonging to ST225 (t003, *n* = 4), ST2883 (t4336, *n* = 2) and ST398 (t011, *n* = 1) did not have the *bla*Z gene and were phenotypically resistant to penicillin.

Among macrolides and lincosamides, 74.4% (*n* = 61) of MRSA isolates showed resistance to erythromycin and clindamycin. Of these, 82% (*n* = 50) had the constitutive erythromycin-clindamycin resistance phenotype, while 18% (*n* = 11) had the inducible erythromycin-clindamycin resistance phenotype. Fifty-nine (72%) isolates were resistant to clindamycin, ciprofloxacin, and erythromycin, most of which belonged to ST225 (*n* = 36) and ST2883 (*n* = 13).

9.1% (*n* = 7) of MRSA isolates belonging to ST1 (t127, *n* = 2), ST45 (t6890, *n* = 2), ST225 (t003, *n* = 1) and ST398 (t011, *n* = 2) were phenotypically resistant to tetracycline. One isolate that belonged to ST225 (t003) lacked antibiotic resistance genes *tet*K and *tet*M and did not correlate with the phenotypic results.

For the aminoglycosides, only 1.3% (*n* = 1; t359, ST97) of isolates showed phenotypic resistance to gentamicin, while the resistant gene *aad*D (*n* = 2; t002, ST5), *ant*(6)-Ia (*n* = 1; t127, ST1), *ant*(9)-Ia (*n* = 52; t002 (*n* = 2), ST5; t003 (*n* = 26), t014 (*n* = 4), t045 (*n* = 5), t1227 (*n* = 1), NT (*n* = 1), ST225; t4336 (*n* = 12), t19843 (*n* = 1), ST2883 and *aph* (3´)-III (*n* = 1, t127, ST1) were determined. Agreement between phenotypic and genotypic susceptibility testing is shown in Table 1.

**Table 1:** Concordance of phenotypic antimicrobial resistance profile with genotypic profile

Clonal complex	Sequence type	SCCmec type	spa type	Phenotypic antimicrobial resistance profile (number of isolates)	Antimicrobial resistance gene (number of isolates)	Total number (%)
CC1	ST1	IVa (2B)	t127	PEN-TET (1)	*bla*Z (1), *tet*K (1)	2 (2.4)
				PEN-CLI-ERY-TET (1)	*bla*Z (1), ant (6)-Ia (1), aph (3¨)-III (1), *erm*C (1), *tet*K (1)	
CC5	ST5	II (2 A)	t002	PEN-CLI-CIP-ERY (2)	*bla*Z (2), *aad*D (2), *ant* (9)-Ia (2), *erm*A (2)	2 (2.4)
		IVa (2B)	t010	PEN (1)	*bla*Z (1)	1 (1.3)
	ST225	II (2 A)	t003	PEN-CLI-CIP-ERY (25)	*bla*Z (21), *erm*A (25)	37 (45.1)
				PEN-CLI-ERY-TET (1)	*bla*Z (1), *erm*A (1), *ant* (9)-Ia (1)	
			t014	PEN-CLI-CIP-ERY (4)	*bla*Z (4), *erm*A (4), *ant* (9)-Ia (4)	
			t045	PEN-CLI-CIP-ERY (5)	*bla*Z (5), *erm*A (5), *ant* (9)-Ia (5)	
			t1227	PEN-CLI-CIP-ERY (1)	*bla*Z (1), *erm*A (1), *ant* (9)-Ia (1)	
			NT	PEN-CLI-CIP-ERY (1)	*bla*Z (1), *erm*A (1), *ant* (9)-Ia (1)	
	ST2883	II (2 A)	t4336	PEN-CLI-CIP-ERY (12)	*bla*Z (10), *erm*A (12), *ant* (9)-Ia (12)	13 (15.9)
			t19843	PEN-CLI-CIP-ERY (1)	*bla*Z (1), *ermA* (1), *ant* (9)-Ia (1)	
CC22	ST22	IV (2B&5)	t022	PEN-CIP-ERY (2)	*bla*Z (2), *erm*C (2)	8 (9.8)
				PEN (1)	*bla*Z (1), *erm*C (1)	
			t790	PEN-CLI-CIP-ERY (4)	*bla*Z (4), *erm*C (4)	
			t11581	PEN-CLI-CIP-ERY (1)	*bla*Z (1), *ermC* (1)	
CC45	ST45	IVa (2B)	t6890	PEN-TET (2)	*bla*Z (2), *tet*K (2)	2 (2.4)
CC97	ST97	IVc (2B)	t359	PEN (12)	*bla*Z (12)	15 (18.3)
				PEN-GEN (1)	*bla*Z (1), *aac* (6´)-*aph* (2¨) (1)	
			t2770	PEN-CLI-CIP-ERY (1)	*bla*Z (1)	
			NT	PEN (1)	*bla*Z (1)	
CC398	ST398	V (5C2&5)	t011	PEN-TET (2)	*bla*Z (1), tetK (1), *tet*M (2)	2 (2.4)

Legend: NT − non typable; PEN – penicillin; CLI – clindamycin; CIP – ciprofloxacin; ERY – erythromycin; GEN – gentamicin; TET – tetracycline

### Virulence genes

**Fig. 1:**
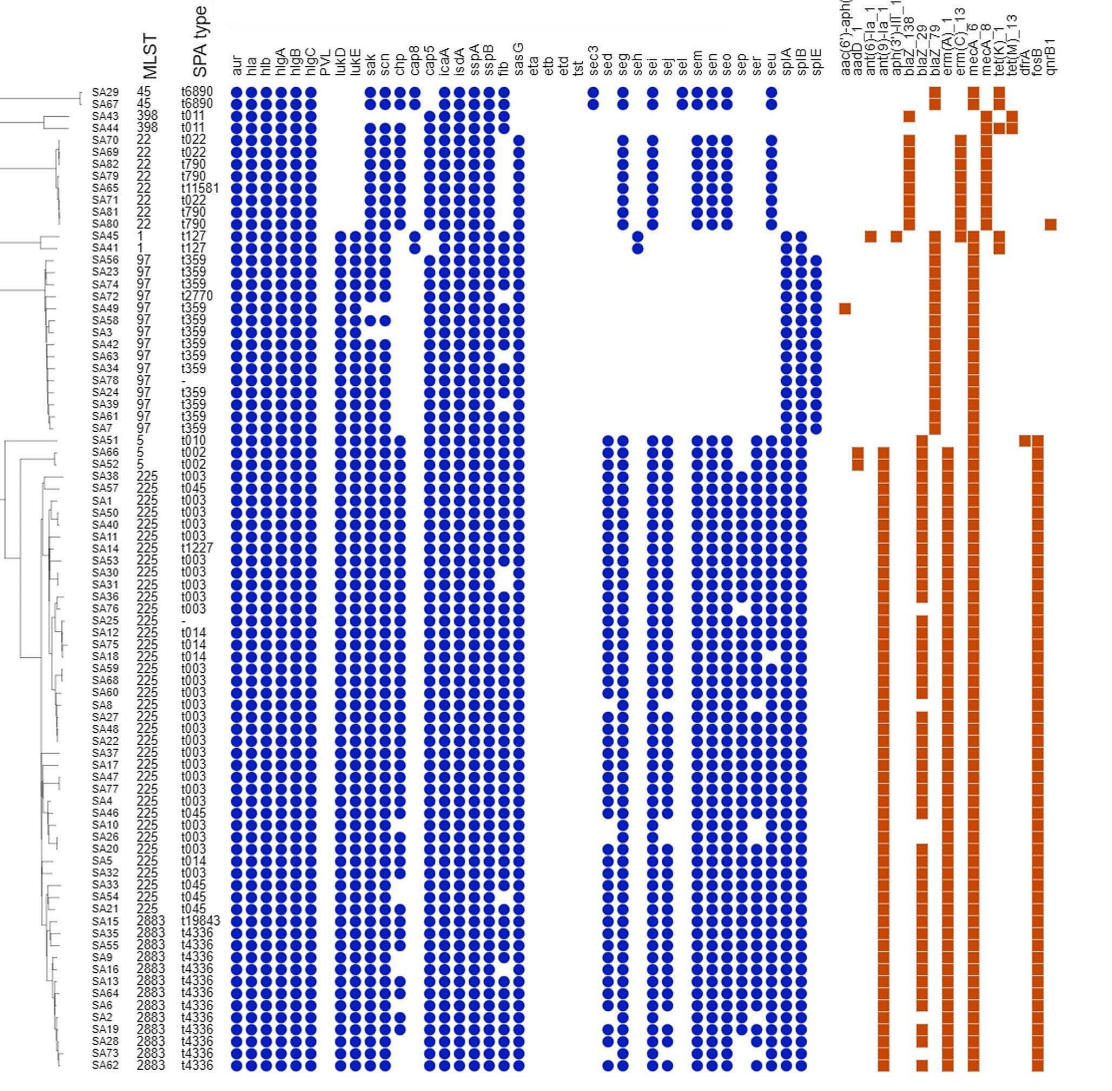
Neighbour joining tree of 82 MRSA isolates, based on cgMLST allelic differences of 1861 alleles. The blue circles and orange squares represent presence or absence of select virulence and antimicrobial resistance genes, respectively

Virulence genes were diverse and correlated with typing characteristics (Fig. 1). Genes encoding aureolysins (*aur*), alpha (*hla*), beta (*hlb*), gamma-hemolysin A, B, C (*hlg*ABC) and leukocidins (*luk*D, *luk*E), were detected at frequencies of 100%, 100%, 100%, 100%, and 85.4% of all MRSA isolates, respectively. *Staphylococcus* complement inhibitor (*scn*) and staphylokinases (*sak*) were detected in 96.3% of all MRSA isolates, whereas no virulence genes were confirmed in three MRSA isolates belonging to ST97 (t359, *n* = 2) and ST398 (t011, *n* = 1). 95.1% MRSA isolates contained the *cap*5 gene, while the *cap*8 gene carried 4 isolates that belonged to ST1 (*n* = 2, *spa* type t127) and ST45 (*n* = 2, *spa* type t6890). The intracellular adhesion gene *ica*A was present in all isolates.

None of the isolates carried Panton-Valentine leukocidin (*luk*SF-PV), exfoliative toxins (*eta, etb, etd*) or toxic shock toxin (*tst*). Many of the isolates carried an enterotoxin or enterotoxin-like genes, including *sec* (*n* = 2), *sed* (*n* = 48), *seg* (*n* = 63), *seh* (*n* = 2), *sei* (*n* = 63), *sej* (*n* = 48), *sel* (*n* = 2), *sem* (*n* = 63), *sen* (*n* = 63), *seo* (*n* = 63), *sep* (*n* = 46), *ser* (*n* = 48), and *seu* (*n* = 62). The majority of isolates (79.3%, *n* = 65) carried ≥ 1 enterotoxin gene, while 17 isolates had no enterotoxin genes. These isolates belonged to ST97, *spa* type t359 (*n* = 13), t2770 (*n* = 1), NT (*n* = 1) and ST398, *spa* type t011 (*n* = 2).

## Discussion

In our study, we phenotypically and genotypically analysed 82 MRSA strains isolated from patients with bloodstream infections in a central region of Slovenia in 2019–2022. This represents approximately one third of Slovenian MRSA isolates submitted to EARS-Net.

In our study, three SCC*mec* types (II, IV, V), eight ST types (ST1, ST5, ST22, ST45, ST97, ST225, ST398, ST2883), six CC complexes (CC1, CC5, CC22, CC45, CC97, CC398) and 16 *spa* types (t002, t003, t010, t011, t014, t022, t045, t127, t359, t790, t1227, t2770, t4336, t6890, t11581, t19843) were identified.

According to SKUOPZ data, MRSA isolates from clinical samples in Slovenia in 2017 were resistant to erythromycin, clindamycin, and ciprofloxacin in 75.2%, 74.3%, and 73%, respectively [10]. In our study we show that the majority of MRSA strains (*n* = 59) were also resistant to multiple antimicrobials (erythromycin, clindamycin, and ciprofloxacin) and belonged to ST225 (*n* = 36), ST2883 (*n* = 13), ST22 (*n* = 7), ST5 (*n* = 2) and ST97 (*n* = 1). MRSA strains (*n* = 20) that were susceptible to erythromycin, clindamycin and ciprofloxacin belonged to ST1 (*n* = 1), ST5 (*n* = 1), ST22 (*n* = 1), ST45 (*n* = 2), ST97 (*n* = 13), and ST398 (*n* = 2). The concordance of phenotype and genotype with the identified resistance gene was 97.8%. Discordant results for penicillin resistance could be due to incorrect reading of the edge of the inhibition zone as fuzzy or sharp according to EUCAST standards [18]. Discrepancies between phenotype and genotype resistance could either be due to the isolates having a nonexpressed resistance gene, plasmid loss, the isolates lacking the resistance gene or conferring other resistance mechanisms. All isolates of our most prevalent ST225-II and ST2883-II carried resistance genes against erythromycin and exhibited phenotypic resistance. Macrolide, ciprofloxacin, and mupirocin resistance phenotypes were described as biomarkers of epidemiological success in a recent study by Baede et al., whereas gentamicin resistance was associated with sporadicity [33]. These observations are consistent with our results.

CC5 (*n* = 53, 64.7%) was the predominant clone identified among MRSA strains and is the most prevalent clone causing HA-MRSA infections in the Western hemisphere [34]. In our study, CC5 included ST5, ST225 and ST2883.

ST225-II, also known as the Rhine Hesse MRSA clone [35] was identified in 37 isolates (45.1%). The most common *spa* type in our study that belonged to ST225-II, t003 (*n* = 26), was also one of the most prevalent *spa* types causing invasive MRSA infections in Europe [7]. According to the study by Kotnik-Kervokijan et al. [13], these *spa* types t003 have been found in Slovenia previously, so ST225-II clone is probably one of the endemic clones circulating in our country. In our study, all ST225-II strains carried inducible resistance by the erythromycin *erm*A gene and the aminoglycoside resistance gene *ant*(9)-Ia, as well as the enterotoxin genes *seg*, *sei*, *sem*, *sen* and *seo*.

ST2883-II was identified in 13 isolates (15.9%), twelve of which belonged to *spa* type t4336, and one to t19843. A novel ST2883 was identified among suspected CA-MRSA in Slovenia in a previous study in 2010 [12]. To our knowledge, no further data are available. All ST2883-II MRSA isolates were resistant to penicillin, erythromycin, clindamycin, and ciprofloxacin, carried *luk*ED genes, enterotoxin genes (*seg, sei, sem, sen, seo, seu*), and haemolysin genes (alpha, beta, gamma). Alleles of sequence types ST225 and ST2883 only differ in a single nucleotide in the gene *yqi*L. Based on pairwise allele differences, some of the isolates belonging to ST225 are more closely related to ST2883 than other isolates belonging to the same ST. The two sequence types are highly similar; of the seven genes used for typing, there is a single nucleotide difference in the *yqi*L gene. ST2883 clone is a novel variant of ST225 and an important clone circulating in Slovenia that causes bloodstream infections.

ST5-II was detected in two isolates (2.4%, *spa* type t002) and is a pandemic strain also known as the New York/Japan clone [35]. In 2011, *spa* type t002 was the fourth most common *spa* type among invasive MRSA in Europe according to Grundmann et al. [7]. Both strains harboured the macrolide resistance gene *erm*A, aminoglycoside resistance gene *aad*D and the virulence-associated genes *sed, seg, sei, sem, sen, seo, ser*, and *seu*. We also detected one (1.3%, t010) MRSA strain belonging to ST5-IV, also known as the Paediatric clone [35]. The strain was resistant to penicillin and carried the *bla*Z gene, the haemolysin genes (alpha, beta, gamma), the *luk*ED gene, and- the enterotoxin genes (*sed, seg, sei, sej, sem, sen, seo, ser*, and *seu)*.

The second most frequent clone was CC97, ST97-IVc, which was identified in 15 isolates (18.3%). Of these, 13 belonged to *spa* type t359, one to *spa* type t2770 and one was non-typable. This clone is usually associated with livestock and rarely with human infections [36, 37]. *Spa* type t359 was previously confirmed in Slovenia among suspected human CA-MRSA isolates in 2014 and 2015 [11]. ST97-IV-t359 has also been found in Bosnia and Herzegovina and in a maternity hospital in Ireland [38, 39]. Although reports of ST97-IV clone are less common in Europe, it is considered an important pathogen in Slovenia. In our study, all ST97 MRSA isolates were resistant to penicillin and cefoxitin and carried the *luk*ED gene. The ST97 isolates were negative for exfoliative toxin genes, toxic shock syndrome toxin genes and enterotoxins genes, similarly to isolates of other major LA-MRSA clones (i.e., CC398 and CC1) [2]. Two ST97 isolates were *sak* and *scn* genes negative, indicating a possible animal origin [1, 2].

The third most frequent clone CC22, ST22-IV was identified in eight isolates (9.8%), of which three belonged to *spa* type t022, four to t790 and one to t11581. ST22-IV is a pandemic strain also known as EMRSA-15 [35]. According to the study by Grundmann et al. the *spa* type t022 was the ninth most common *spa* type among invasive MRSA in Europe in 2011 [7]. All isolates belonging to ST22-IV in our study were resistant to penicillin, 25% to ciprofloxacin, erythromycin and clindamycin, and harboured haemolysin (alpha, beta, gamma) and enterotoxin genes (*seg, sei, sem, sen, seo*, and *seu)*.

We have also identified other clones associated with LA-MRSA: two isolates belonged to ST1-IVa (2.4%, *spa* type t127) and two belonged to ST398-V (2.4%, *spa* type t011). All strains were resistant to penicillin, tetracycline, 25% to erythromycin and clindamycin. *spa* type t127 carried the *luk*ED gene and the enterotoxin gene *sek*. The *sak* and the *scn* genes were negative in one strain, *spa* type t011, ST398, indicating a possible animal origin [1, 2]. None of the ST398-V-t011 strains had genes associated with virulence (enterotoxins, Pantone-Valentine leukocidin). According to our previous study among suspected CA-MRSA [11, 12] and the Kotnik-Kervokijan study [13], the ST398 clone has spread in the community and healthcare settings throughout the country. Currently, the number of severe infections caused by ST398 remains low in a central region of Slovenia. Larsen and colleagues reported an increased number of LA-MRSA ST398 bacteremias in Denmark, where most patients lived in rural areas but had no contact with livestock. The researchers predicted that the number of severe infections and deaths will increase if LA-MRSA ST398 is allowed to spread in the general population [39].

The final CC45 clone, ST45-IV was identified in two isolates (2.4%, *spa* type t6890) [35]. Both strains were resistant to penicillin and tetracycline, and they carried haemolysin (alpha, beta, gamma) and enterotoxin genes (*sec, e.g., sei, sel, sem, sen, seo*, and *seu)*.

Surprisingly, we did not find any *mec*C positive clone, otherwise found in previous studies, in which *mec*C positive strains were detected both in asymptomatic carriers and also in clinical samples (sputum, blood culture) [11, 12, 14].

The virulence factors of *S. aureus* play an important role during pathogenesis [1–3]. Overall, in our study, all MRSA isolates harboured exotoxin genes (*hla, hlb* and *hlg)*, adhesin (*icaA*) and enzymes (*sspA, sspB*) which may facilitate invasion and immune evasion. We also confirmed that over 50% of bloodstream MRSA isolates encode *seg, sei, sem, sen, seo* and / or *seu* [40]. Surprisingly, all isolates that belonged to ST97 had none of the enterotoxin genes, while two isolates that belonged to ST1 harboured only *seh*. According to the Bennett and Thomsen study the leucocidin toxin genes *luk*ED are common across *S. aureus* lineages, with approximately 87% clinical isolates carrying *luk*ED [41]. A similar percentage (85.4%) was found in our study, and only MRSA strains that belonged to ST22, ST45 and ST398 were *luk*ED negative.

The main limitation of our study is the incomplete access to medical data and data on possible patient transfers between hospitals. Therefore, we could not investigate possible transmission events or investigate correlation over time. Additionally, two isolates in our study were non-typeable by *spa* typing. Due to limitations of short-read sequencing when it comes to repetitive regions, as well as possible low sequencing coverage in this region, *spa* types could not be reliably determined, because multiple *spa* repeats were assembled, or the 5’ or 3’ signatures were missing. *Spa* types were considered reliable when only one repeat sequence was recognised and the 5’ and 3’ signatures were found in the correct positions, as was the case for other isolates. For further studies, long-read sequencing could be employed to resolve these limitations. Like other epidemiologic studies, our study is also limited by the selected isolates.

## Conclusions

In summary, we present diverse lineages of MRSA causing bacteraemia in a central region of Slovenia. Currently, the new genetic MRSA lineage ST2883 plays an important role in the spread in the Slovenian population. Clonal replacement is frequent, and the identification of emerging lineages should be monitored in the future to detect changes in the molecular epidemiology of MRSA and guide interventions to reduce the burden of MRSA in Slovenia.

### Electronic supplementary material

Below is the link to the electronic supplementary material.

Supplementary Material 1

## Data Availability

The generated raw reads of all isolates were submitted to the European nucleotide archive under the study accession number PRJEB66124.
